# Adaptive training load optimization for sprinters via offline reinforcement learning and multi-modal physiological feedback

**DOI:** 10.3389/fpsyg.2026.1902098

**Published:** 2026-10-28

**Authors:** Xinhe Hu, Linjun Liu, Yuting Pei

**Affiliations:** 1 College of Physical Education and Health, Guangxi Normal University, Guilin, Guangxi, China; 2 College of Sports Medicine, Wuhan Sports University, Wuhan, China; 3 Faculty of Pharmacy, Lincoln University College, Petaling Jaya, Selangor, Malaysia; 4 Faculty of Information Measurement and Biotechnical Systems, Saint Petersburg Electrotechnical University “LETI”, St. Petersburg, Russia

**Keywords:** heart rate variability, off-policy evaluation, reinforcement learning, retrospective cohort study, sprint performance, training load, wearable sensors

## Abstract

Elite sprint training requires daily adjustment of training load to balance performance adaptation and fatigue, yet physiological monitoring does not necessarily translate into a specific next-session prescription. This study formalizes daily sprint training prescription as a finite-horizon Markov Decision Process and develops an adaptive load-optimization framework that couples multi-modal physiological feedback with offline reinforcement learning (RL). A 19-dimensional state fusing autonomic, neuromuscular, endocrine, subjective, and load-aggregate signals is encoded by a dual-tower network [a one-dimensional convolutional neural network–Transformer temporal branch and a multi-layer perceptron (MLP) static branch] over an action space of 62 admissible training-type/intensity/volume combinations. A composite reward penalizes overreaching while rewarding short-term gain, longitudinal adaptation, and acute-to-chronic workload ratio conformity. The policy is learned by conservative off-policy training on retrospective data, evaluated through the Fitted Q-Evaluation (FQE) and Doubly Robust (DR) estimators, and presented to coaches as the three leading candidate actions, with SHapley Additive exPlanations (SHAP) values used to explain the model's preferences among the candidate actions. In a 12-week single-center retrospective comparison of 70 national-level sprinters (35 men and 35 women; 100 m personal best (PB) ≤ 11.20 s for men or ≤ 12.50 s for women), grouped by whether their squad had adopted the tool (*n* = 35 RL-guided) or continued classical block periodisation (*n* = 35 control), the RL-guided cohort demonstrated between-cohort differences of +0.240 s in 100 m [95% confidence interval (CI) +0.128 to +0.352; *p* < 0.001, *d* = 1.00], +0.104 s in 30 m (*p* < 0.001, *d* = 1.00) and +2.17 cm in countermovement jump (*p* < 0.001, *d* = 1.01). Zero algorithmically defined sustained multi-indicator fatigue/load events were observed in the RL-guided cohort vs. seven in the control cohort (1.43 per 1,000 athlete-days); these events should not be interpreted as clinically diagnosed overtraining syndrome. Off-policy evaluation yielded exploratory point estimates whose precision remains unknown because uncertainty intervals were not calculated. These observational findings describe associations with an RL-guided, coach-in-the-loop workflow and do not establish a causal effect of the RL policy.

## 1 Introduction

Short-distance sprint events (100 m, 200 m, and 400 m) place an extreme and repeated demand on the neuromuscular, anaerobic-glycolytic, and central nervous systems, with elite performance now improving by only a few hundredths of a second per season (Spencer et al., 2005; Laursen and Jenkins, 2002). At this level, the marginal returns from further increasing total training volume are negligible, and competitive gains depend instead on how precisely the daily training stimulus is matched to the athlete's transient state of readiness (Halson, 2014; Bourdon et al., 2017). Decades of periodisation theory, from Matveyev's original macrocycle framework to Issurin (2010)'s block periodisation and Kiely (2012)'s critical re-examination of the empirical foundations of periodisation, have provided the conceptual scaffold for weekly and monthly planning; yet the day-to-day prescription remains a coach-driven heuristic that synthesizes template plans, anecdotal observation, and informal physiological cues (Akenhead and Nassis, 2016; Bartlett et al., 2017).

A parallel revolution in athlete monitoring has produced an unprecedented volume of multi-modal, high-frequency physiological data. Heart rate variability (HRV) trends, derived from chest-strap or wrist optical sensors, are now widely accepted as a non-invasive marker of autonomic recovery (Plews et al., 2013; Buchheit, 2014). The session rating of perceived exertion (sRPE), introduced by Foster et al. (2001), remains the de facto internal-load scale across team and individual sports (Impellizzeri et al., 2005; McLaren et al., 2018), whereas wearable inertial sensors, Global Positioning System (GPS) data, and electromyographic sensors enable the continuous quantification of external load (Seshadri et al., 2019; Teixeira et al., 2021). The acute-to-chronic workload ratio (ACWR), proposed by Gabbett (2016), has been used as a workload-monitoring indicator, although its predictive validity in isolation has been challenged and it is best interpreted alongside other physiological and subjective measures (Saw et al., 2016; Bartlett et al., 2017; Windt and Gabbett, 2017; Soligard et al., 2016). Subjective wellness questionnaires capture dimensions that no objective sensor reaches (Saw et al., 2016). Although such monitoring can inform the assessment of readiness and workload, translating these measurements into a specific subsequent training prescription remains a distinct decision-making problem. In sprint training, this requires selecting a next-session action from heterogeneous, changing physiological information while accounting for the athlete's response to previous training and the coach's final judgement.

Machine learning has begun to bridge this gap on the predictive side. Recent reviews by Claudino et al. (2019) and Teixeira et al. (2021) document a proliferation of supervised models for injury-risk classification and performance forecasting in team sports, including GPS-driven injury forecasting (Rossi et al., 2018) and ensemble approaches in young athletes (Jauhiainen et al., 2021). Big-data tactical analysis in elite soccer (Rein and Memmert, 2016) has further illustrated the potential of data-driven inference in sport contexts. These approaches primarily estimate outcomes such as injury risk or performance; prediction alone does not specify which training action should follow. Sequential decision-making methods, including reinforcement learning (RL), provide a formal framework for relating an evolving state to a sequence of actions and their longer-term outcomes. The Markov Decision Process formulation (Puterman, 2014; Sutton and Barto, 2018) is therefore relevant to daily training-load decisions, which involve physiological state, structured training choices, and trade-offs between performance adaptation and fatigue. Applications of RL in healthcare provide examples of learning from observational records and evaluating candidate policies, but their clinical settings differ from the small, safety-sensitive sprint-training context (Yu et al., 2023).

Yet three structural obstacles have limited the application of RL to real sprint training. First, sprint cohorts are small, and the athletes are physically vulnerable; random on-policy exploration is ethically and practically infeasible, motivating offline learning from existing records with conservative policy constraints (Arulkumaran et al., 2017). Second, the relevant state is multi-modal, multi-timescale, and heterogeneous, combining continuous time series with daily ratings and less frequent laboratory tests. Third, coaches and athletes require recommendations that can be reviewed and adapted, so a practical system must support human oversight and provide interpretable candidate actions (Kellmann et al., 2018). Together, these constraints make the problem more than one of predicting readiness: a useful decision-support approach must represent changing athlete state, learn from logged actions without unsafe exploration, and leave the final prescription under coach oversight.

This study describes and retrospectively evaluates an offline-RL-guided, coach-in-the-loop workflow for sprint-training decision support. The framework comprises four components. (i) A *dual-tower state encoder* processes the last 14 days of 12 physiological and performance signals through a one-dimensional convolutional neural network and Transformer encoder, whereas a parallel multi-layer perceptron handles static features; their outputs form a 19-dimensional state vector. (ii) A *structured discrete action space* contains 62 admissible combinations of training type, intensity, and volume, obtained by filtering biomechanically implausible combinations from the full Cartesian product. (iii) A *composite reward* weights short-term performance change, longitudinal adaptation, ACWR conformity and multi-indicator fatigue/load events (Meeusen et al., 2013; Banister et al., 1975). (iv) A *three-stage offline learning pipeline* trains a conservatively regularized Actor-Critic on retrospective data, evaluates it through the Fitted Q-Evaluation, Doubly Robust, and Per-Decision Importance Sampling (PDIS) estimators, and presents the top-three candidate actions with SHAP attributions for coach review. The retrospective comparison includes 70 national-level sprinters from two squads, one using the decision-support tool as part of its routine coaching workflow and one following classical block periodisation. Because cohort membership was determined by squad and the coach could accept, modify, or reject recommendations, the comparison evaluates the association with the overall workflow and cannot isolate the effect of the RL policy itself. Primary outcomes were 100 m time, 30 m time, and countermovement jump height; secondary outcomes included workload and algorithmically defined multi-indicator fatigue/load events. Accordingly, the findings are interpreted as observational associations that motivate prospective evaluation, rather than as evidence of a causal treatment effect. The study does not include a matched comparison with simpler rule-based, HRV-guided or supervised adaptive alternatives and therefore does not establish the incremental value of the proposed RL architecture over such approaches.

## 2 Related work

### 2.1 Training-load monitoring and periodisation

The quantification of athletic training load has converged on a small number of internal-load [sRPE, training impulse (TRIMP)], HRV-based indices and external-load (distance, accelerations, ground reaction force, velocity zones) summaries, codified in the consensus statement by Bourdon et al. (2017) and refined by subsequent meta-analytic work (McLaren et al., 2018). ACWR has been used as a workload-monitoring indicator, although its predictive value in isolation has been challenged and it is best interpreted alongside HRV trends and subjective wellness (Gabbett, 2016; Saw et al., 2016; Bartlett et al., 2017; Windt and Gabbett, 2017). Practitioner surveys reveal that despite this evidence base, monitoring decisions in elite football remain largely subjective, with coach experience influencing how monitoring information is used (Akenhead and Nassis, 2016).

Periodisation theory provides the broader scaffold for organizing training across weeks and months. The Banister fitness-fatigue model (Banister et al., 1975) casts performance as the difference between accumulated fitness and accumulated fatigue, each with its own time constant; this remains a touchstone for quantitative reasoning about training adaptation. Block periodisation has been advocated by Issurin (2010) for time-pressed elite athletes, whereas Kiely (2012) has argued that the empirical evidence for any specific periodisation paradigm is weaker than its widespread adoption suggests. The athlete monitoring cycle (Kellmann et al., 2018) and the International Olympic Committee consensus on load and injury risk (Soligard et al., 2016) recommend an individualized, feedback-driven approach, but do not prescribe a formal decision procedure for choosing each day's training action. Thus, monitoring and periodisation provide useful information and planning structure, whereas the translation of changing athlete state into a specific next-session prescription remains an open practical challenge.

### 2.2 Physiology of sprint and interval training

Sprint events impose repeated maximum-intensity efforts with incomplete recovery, eliciting both phosphocreatine and anaerobic glycolytic adaptations alongside neuromuscular potentiation (Spencer et al., 2005). High-intensity interval training, summarized in reviews by Laursen and Jenkins (2002) and Buchheit and Laursen (2013), demonstrates that the optimal stimulus depends on a complex interplay of work-bout duration, intensity, recovery type, and individual training status. Polarized training intensity distributions have been examined in endurance athletes (Seiler, 2010), providing further evidence that the timing of intensity can matter alongside total volume. For sprint training, these findings motivate attention to the timing and sequence of training stimuli, but do not by themselves provide an individualized rule for selecting the next session from multi-modal monitoring data.

### 2.3 Reinforcement learning in clinical and physiological settings

RL formalizes sequential decisions in which actions are selected according to the current state and evaluated in relation to subsequent outcomes. Standard texts describe the underlying Markov Decision Process framework (Sutton and Barto, 2018; Puterman, 2014), while methodological surveys review deep RL and its application to healthcare settings (Arulkumaran et al., 2017; Yu et al., 2023). Healthcare applications have explored policy learning from observational records, conservative learning objectives, and off-policy evaluation. These studies provide methodological precedents, but their treatment decisions, patient populations, and safety considerations differ from daily sprint-training prescription. The relevance to this study is therefore methodological: offline policy learning and evaluation offer tools for studying candidate training decisions from existing records, but do not resolve the domain-specific issues of athlete-level data, limited action coverage and coach oversight.

### 2.4 Machine learning in sport-science applications

A systematic review by Claudino et al. (2019) surveys artificial intelligence (AI) applications to injury-risk assessment and performance prediction in team sports. Rossi et al. (2018) demonstrated injury forecasting in soccer using GPS training data and supervised machine learning, while Jauhiainen et al. (2021) applied classifiers to injury-risk factors in young team-sport athletes. Multi-modal sensors and big-data analytics have also been examined in elite soccer tactical analysis (Rein and Memmert, 2016) and football load monitoring (Teixeira et al., 2021). Ahmadian and Rostami (2024) studied transformer-based methods alongside RL in physical-activity recognition. Collectively, this literature demonstrates progress in monitoring, prediction, and activity recognition, whereas it leaves open how a specific next-session prescription can be derived from longitudinal performance and physiological information under safety constraints. This study addresses this question as a retrospective evaluation of an offline-RL-guided coaching workflow; because the comparison is observational and cohort membership is aligned with squad membership, it does not isolate the causal contribution of RL from coach decisions or other squad-level factors.

## 3 Methods

### 3.1 Overall pipeline

Figure 1 summarizes the decision-support workflow characterized in this study. On any given day *t*, multi-modal sensors and laboratory tests produce physiological and performance signals (stage 1). These are aggregated and normalized into a 19-dimensional state vector *s*_*t*_ by the dual-tower encoder (stages 2–3). The offline-RL policy proposes the three highest-valued admissible actions, each annotated with SHAP-based feature attributions and an ACWR-based risk indicator (stage 4). In the RL-guided cohort, the coach reviewed, accepted, modified, or rejected these recommendations as part of routine coaching practice. The comparison reported here is retrospective and observational; it evaluates the cohort-level association with this coach-in-the-loop workflow and does not isolate the effect of the RL policy. Figure 2 details the neural network architecture, including the input-stream encoding, the dual-tower state encoder, the conservatively regularized Actor-Critic head, the reward and Bellman-update block, and the safety gate.

**Figure 1:**
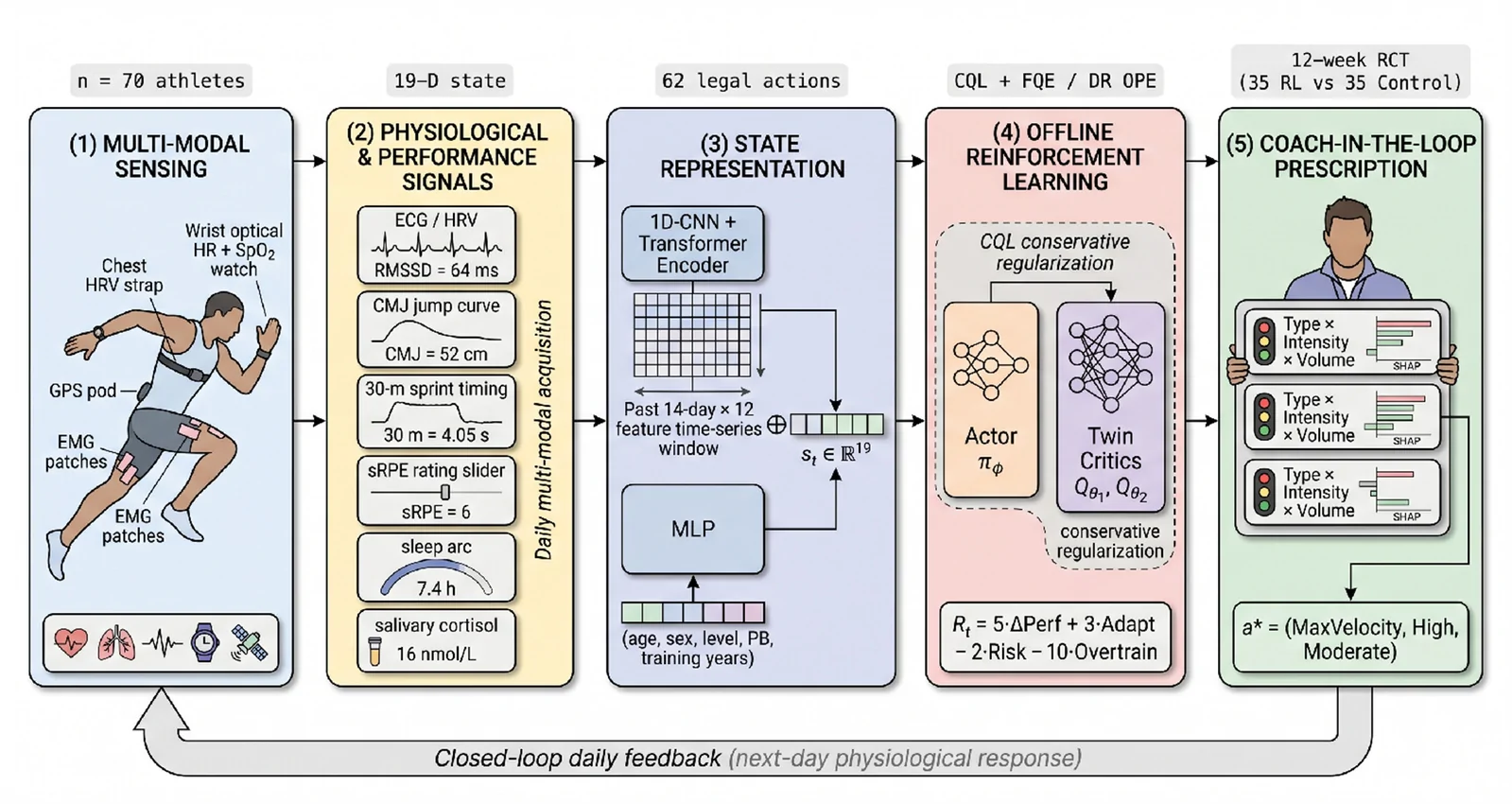
Overview of the closed-loop adaptive training-load optimization pipeline. Multi-modal sensing on the athlete (stage 1) produces daily physiological and performance signals (stage 2) that are fused into a 19-dimensional state vector by a dual-tower encoder (stage 3). An offline RL policy with conservative regularization proposes candidate training prescriptions (stage 4), which are reviewed by a coach with SHAP-based explanations as part of routine practice in the RL-guided cohort and feedback the next-day response (stage 5). The 12-week retrospective observational two-cohort comparison (*n* = 70: 35 RL-guided vs. 35 Control) is reconstructed from the daily records produced by this pipeline.

**Figure 2:**
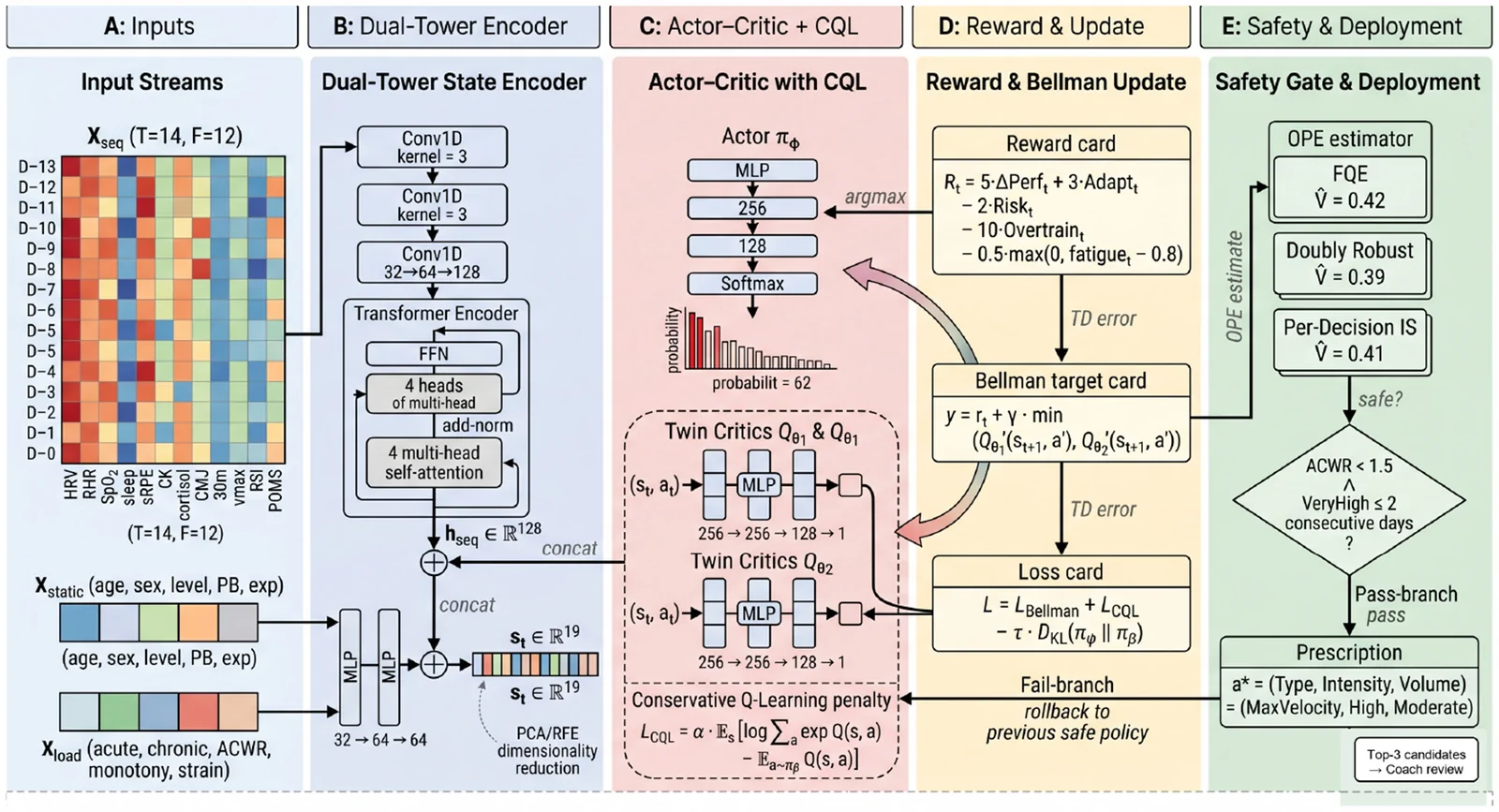
Detailed neural-network architecture. **(A)** The input comprises a 14 × 12 time-series tensor, a 5-dimensional static-feature vector, and a 5-dimensional load-aggregate vector. **(B)** A dual-tower state encoder combining a one-dimensional convolutional neural network (1D-CNN) with a Transformer encoder (4-head self-attention) for the temporal branch and a multi-layer perceptron for the static branch produces the 19-dimensional state vector *s*_*t*_. **(C)** An Actor π_ϕ_ outputs a softmax distribution over 62 discrete actions; twin Critics *Q*_θ_1__, *Q*_θ_2__ are jointly regularized by a conservative penalty that pushes down *Q*-values on out-of-distribution actions. **(D)** The composite reward drives a clipped-double-*Q* Bellman update with the loss $L=L_{\text{Bellman}}+L_{\text{Cons}}-\tau D_{\text{KL}}\left(\pi_{\phi }||\pi_{\beta }\right)$. **(E)** Three off-policy estimators (Fitted Q-Evaluation, Doubly Robust, Per-Decision Importance Sampling) and a hard safety gate based on ACWR and consecutive Very-High days filter the policy output before the top-three are passed to the coach.

### 3.2 Study design and participants

We conducted a single-center, two-cohort, retrospective observational comparative study. The dataset window comprised 20 consecutive weeks of routine training and monitoring records, organized as a 14-day individual baseline-calibration block, a 28-day observation block, a 12-week mesocycle structure (study weeks 7–18) of accumulation, intensification, and competition, and a 14-day retest block. The 70 sprinters whose anonymised records constituted the dataset were drawn from two training squads at the same national-level training center, one of which had adopted the offline-RL decision-support tool as part of its routine coaching practice during the dataset window (RL-guided cohort, *n* = 35), whereas the other had continued with classical block periodisation under the same head-coach group (control cohort, *n* = 35). The investigators did not impose a prospective intervention or alter training prescriptions. All daily training actions were selected by the athletes' usual coaching staff according to their established practice. In the RL-guided cohort, recommendations could be accepted, modified, or rejected by the coach; therefore, cohort membership represents exposure to the overall coach-in-the-loop workflow rather than to a uniformly delivered RL prescription. Reporting followed the principles of the Strengthening the Reporting of Observational Studies in Epidemiology (STROBE) statement for observational cohort studies (cohort flow diagram: Supplementary Figure S1). The analysis used anonymised retrospective monitoring data with no additional intervention beyond the athletes' standard training routine; accordingly, the Institutional Review Board of Guangxi Normal University granted ethics exemption under Article 32 of the *Measures for Ethical Review of Life Sciences and Medical Research Involving Human Subjects* (2023) (approval no. GXNU-EC-EXEMPT-2026-0215, dated 15 February 2026). The protocol complied with the Declaration of Helsinki.

Inclusion criteria were: (i) age 18–28 years; (ii) certified national-level (Grade II) or higher with a 100 m personal best ≤ 11.20 s (male) or ≤ 12.50 s (female); (iii) free from major injury (with no fracture, ligament rupture, or surgery during the preceding 6 months) at the start of the dataset window; (iv) written informed consent for secondary use of the de-identified records. Exclusion criteria covered active pharmacotherapy affecting cardiovascular or metabolic function, acute infection within the 30 days preceding the dataset window, and incomplete wearable monitoring records during the dataset window. Sample-size considerations followed Cohen (1988)'s framework with a target between-cohort Cohen's *d* = 0.6 on the primary outcome, α = 0.05 and 1−β = 0.80, yielding *n* = 30 per cohort; allowing for incomplete-record exclusion we analyzed 70 athletes (35 per cohort), with sex and personal best level used as descriptive stratification variables following the recommendations of Hopkins and colleagues for sports-science studies (Hopkins et al., 2009). Athletes were not randomly assigned to cohorts; cohort membership reflected the training squad an athlete belonged to during the dataset window. Because intervention status was aligned with squad membership, differences between cohorts cannot be attributed specifically to the RL policy, and residual squad-level confounding cannot be excluded.

### 3.3 Data acquisition

Daily data collection followed the multi-modal monitoring philosophy summarized in the consensus statement by Bourdon et al. (2017) and the wearables review by Seshadri et al. (2019), and comprised three streams. (a) *Continuous wearable signals*: morning HRV, quantified as the root mean square of successive differences (RMSSD), and resting heart rate (HR) from a chest strap, processed using the corrections recommended by Plews et al. (2013) and Buchheit (2014); peripheral oxygen saturation (SpO_2_) from a wrist optical sensor; sleep duration and quality from an actigraphy wristband; during training sessions, GPS-derived running velocity, surface electromyography (EMG) of the rectus femoris and biceps femoris during sprint efforts, and ground reaction force during force-plate jumps. (b) *Performance tests* on test days T0–T6 (study days 0, 14, 42, 70, 98, 126, and 140): 30 m sprint timing with photoelectric gates, 100 m race simulation, countermovement jump (CMJ) height with a force plate, vertical-stiffness-derived Reactive Strength Index (RSI), and maximal velocity from the 30–60 m segment. (c) *Daily logs*: session sRPE on the 0–10 scale of Foster et al. (2001), training duration and distance, Profile of Mood States (POMS)-Fatigue subscale, wellness score (Saw et al., 2016), and every week salivary cortisol and serum creatine kinase (CK). All records had been collected as part of the athletes' routine monitoring during the dataset window and were not modified for this study.

All sensor streams were time-aligned to Coordinated Universal Time (UTC) plus local offset and indexed by athlete ID, calendar date, and session ID. Missing data affecting up to 10% of observations were handled using forward filling from individual baselines and mean imputation; sessions with >10% missing data for critical variables were flagged and excluded from supervised state inference but retained as terminal states. Outliers were removed using a Hampel filter [median ±3 median absolute deviation (MAD)]. HRV RR-interval series were corrected for ectopic beats; EMG signals were band-pass filtered at 20–450 Hz with a 50 Hz notch.

### 3.4 Markov decision process (MDP) formulation

We model daily prescription as a finite-horizon Markov Decision Process M=〈S,A,P,R,γ〉 with horizon *H* = 140 days (the full study window) and discount factor γ = 0.95, following the canonical formulation of Puterman (2014) and Sutton and Barto (2018).

#### 3.4.1 State space

The state vector $s_{t}\in {\mathbb{R}}^{19}$ at the morning of day *t* encodes the athlete's current readiness across seven physiological-and-performance categories: autonomic (HRV-RMSSD, resting heart rate, SpO_2_), endocrine (salivary cortisol), muscle-damage (creatine kinase), subjective (POMS-Fatigue, wellness, sleep hours, sleep quality), neuromuscular (CMJ height, RSI), performance (30 m time, peak velocity), and load-aggregate (acute load, chronic load, ACWR, monotony, strain) plus a composite fatigue index. All variables are individual-baseline *Z*-scored, in line with the individualized internal-load reasoning recommended by Bartlett et al. (2017).

#### 3.4.2 Action space

An action *a* = (Type, Intensity, Volume) specifies the daily prescription. Type ∈{Rest, Recovery, Technique, Strength, Acceleration, Max Velocity, Speed Endurance}, Intensity ∈{Low, Moderate, High, Very-High} with sRPE midpoints {2.5, 4.5, 6.5, 8.5}, and Volume ∈{Low, Moderate, High}. The Cartesian product yields 84 combinations from which we remove biomechanically implausible options (e.g., *Rest + High, Recovery + Very-High, Acceleration + Very-High + High Volume*), retaining 62 admissible discrete actions. This catalog covers the principal training types reported in elite sprint and high-intensity interval training (HIIT) literature (Laursen and Jenkins, 2002; Buchheit and Laursen, 2013; Spencer et al., 2005).

#### 3.4.3 Reward function

The composite per-step reward jointly captures performance, adaptation, risk, and a study-defined sustained multi-indicator fatigue/load event:


$$\begin{array}{c} \begin{array}{lll} R_{t} & = & \alpha_{1}\Delta {\text{Perf}}_{t}+\alpha_{2}{\text{Adapt}}_{t}-\alpha_{3}{\text{Risk}}_{t}-\alpha_{4}1_{{\text{FatigueLoadEvent}}_{t}} \end{array}\\ \begin{array}{ll} - & \alpha_{5}\max(0,{\text{fatigue}}_{t}-0.8), \end{array} \end{array}$$ (1)

where ΔPerf_*t*_ is the day-on-day relative improvement of 30 m time and CMJ vs. individual baseline, Adapt_*t*_ is the weekly improvement of the composite fatigue index following the Banister fitness-fatigue logic (Banister et al., 1975), Risk_*t*_ = **1**[ACWR_*t*_>1.5or HRV downtrend≥3 d] aligns with the workload–injury etiology model (Windt and Gabbett, 2017; Gabbett, 2016), and **1**_FatigueLoadEvent_*t*__ flags a study-defined sustained multi-indicator fatigue/load event, not a clinical diagnosis of overtraining syndrome. It requires three or more of the following indicators: POMS-F >22, ACWR >1.4, CK >3.5 × baseline, HRV < 0.7 × baseline, and latent fatigue >0.82, sustained for three consecutive days. The weights (α_1_, …, α_5_) = (5, 3, 2, 10, 0.5) were obtained via a three-round Delphi procedure with three certified strength and conditioning coaches and were subsequently refined by Inverse Reinforcement Learning on observation-period data.

#### 3.4.4 Transitions

The transition kernel *P*(*s*_*t*+1_|*s*_*t*_, *a*_*t*_) is learned empirically from the observational dataset; for off-policy evaluation we use the empirical distribution directly without an explicit model.

### 3.5 Dual-tower state encoder

The state encoder $\phi :\left(X_{\text{seq}},X_{\text{static}},X_{\text{load}}\right)\to s_{t}\in {\mathbb{R}}^{19}$ comprises two branches. The temporal branch processes a 14 × 12 window of the last 14 days of 12 daily features (HRV, resting HR, SpO_2_, sleep, sRPE, CK, cortisol, CMJ, 30 m, peak velocity, RSI, POMS) through three causal 1D-CNN blocks (kernel size 3, channels 32 → 64 → 128, layer normalization, GELU activation) followed by a Transformer encoder of two blocks with four heads of multi-head self-attention and a feed-forward dimension of 256, producing a 128-dimensional embedding *h*_seq_. The choice of Transformer over plain recurrence is motivated by recent demonstrations that self-attention preserves long-range physiological dependencies even with limited training data (Ahmadian and Rostami, 2024). The static branch processes the 5-dimensional vector *X*_static_ = (age, sex, level, PB, training years) through a 32 → 64 → 64 multi-layer perceptron, yielding $h_{\text{stat}}\in {\mathbb{R}}^{64}$. The two embeddings are concatenated with the 5-dimensional load-aggregate vector *X*_load_ [acute load, chronic load, ACWR, monotony, strain (Bourdon et al., 2017; McLaren et al., 2018)], projected to 32 dimensions, and reduced to the final 19-dimensional state through a recursive feature elimination plus principal component analysis pipeline that retains 95% of variance while ensuring that the autonomic, neuromuscular, and load categories are each represented by at least three features.

### 3.6 Offline RL training

We train an Actor-Critic policy with twin clipped-double *Q*-networks following the conservative offline RL formulation that adds a penalty discouraging high *Q*-value attribution to out-of-distribution actions, building on the double-*Q* correction of Van Hasselt et al. (2016) and the continuous-control framework of Lillicrap et al. (2015). Each critic *Q*_θ_*k*__, *k*∈{1, 2} is a 256 → 256 → 128 → 1 multi-layer perceptron with layer normalization and dropout 0.1. The actor π_ϕ_ is a 256 → 256 → 128 network with a softmax over 62 actions. The conservative training objective is given in Equation 2:


$$\begin{array}{lll} L_{\text{Cons}}\left(\theta_{k}\right) & = & \alpha E_{s\sim D}\left[\log\underset{a\in A}{\sum }\exp Q_{\theta_{k}}\left(s,a\right)-E_{a\sim \pi_{\beta }\left(\cdot |s\right)}Q_{\theta_{k}}\left(s,a\right)\right] \end{array}$$

$$\begin{array}{ll} + & L_{\text{Bellman}}\left(\theta_{k}\right), \end{array}$$ (2)

where π_β_ is the empirical behavior policy estimated by behavior cloning on the observational data and $L_{\text{Bellman}}$ is the squared temporal-difference (TD) error with the clipped-double target $y=r_{t}+\gamma \underset{k}{\min}Q_{\theta_{k}^{\prime }}\left(s_{t+1},a^{\prime }\right)$. The actor is updated by maximizing $E_{s,a\sim \pi }Q_{\bar{\theta }}\left(s,a\right)-\tau D_{\text{KL}}\left(\pi_{\phi }||\pi_{\beta }\right)$ with KL weight τ = 0.5 to prevent excessive drift from the data distribution (Arulkumaran et al., 2017). Hyperparameter α was selected on validation by sweeping {0.5, 1, 2, 5}; we report α = 2 unless otherwise noted. Training used the Adam optimiser with learning rate 3 × 10^−4^, batch size 256, target-network soft update coefficient 0.005, and ran for 5 × 10^5^ gradient steps on a single NVIDIA RTX A6000. The full pseudocode is given in Algorithm 1.

Algorithm 1 Conservative offline actor-critic training for adaptive training-load optimization. 
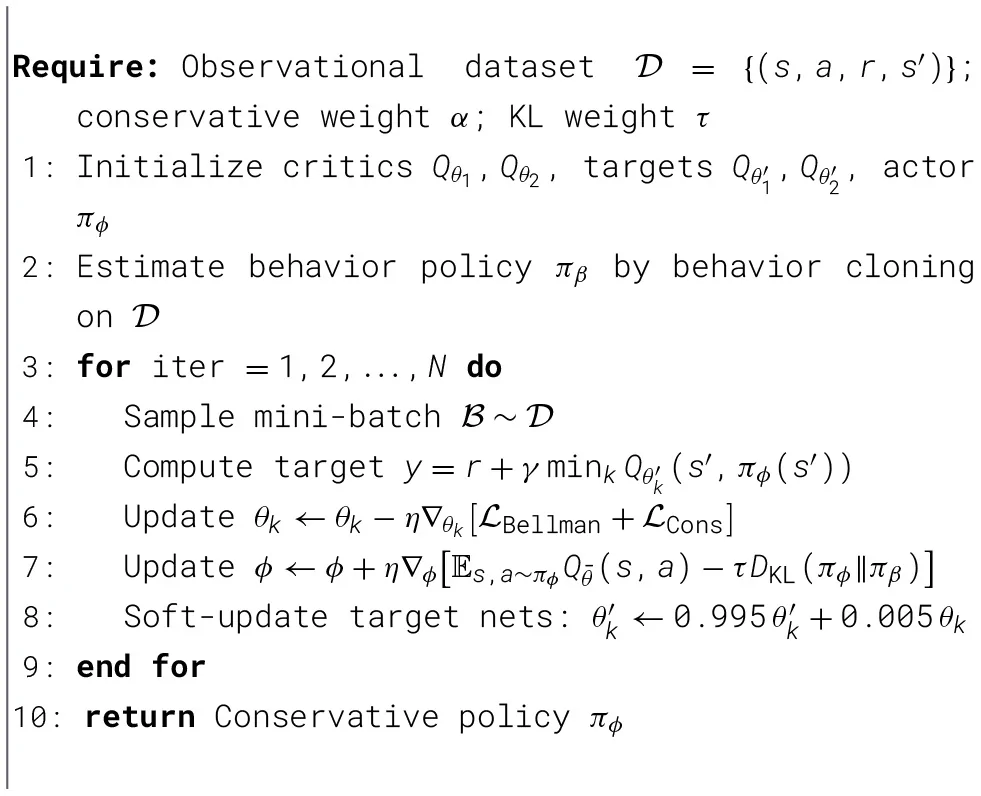

### 3.7 Off-policy evaluation and safety gate

To characterize π_ϕ_ on the cohort data, we estimated its value via three complementary off-policy estimators, mirroring practice in clinical RL (Yu et al., 2023): Fitted Q-Evaluation trained on a held-out 15% of athletes, Doubly Robust combining the Fitted Q-Evaluation (FQE) value function with importance-sampled advantage corrections, and Per-Decision Importance Sampling as a model-free point estimator. The held-out split was performed at the athlete level so that records from a given athlete were not divided across the training and evaluation partitions. No athlete-level bootstrap confidence intervals or other uncertainty intervals were calculated for the off-policy evaluation (OPE) estimates. Their precision is therefore unknown, and they are reported as exploratory point estimates. Agreement among the estimators does not quantify uncertainty or establish superiority over the behavior-policy reference.

The policy was retained for cohort-level analysis only if all three estimators agreed within ±10%, the FQE-estimated value strictly exceeded that of the behavior policy, and the simulated rate of safety-rule violations [ACWR >1.5 or Very-High intensity on >2 consecutive days (Soligard et al., 2016; Windt and Gabbett, 2017)] was below 5% of athlete-days. These were operational screening criteria based on point estimates and prespecified rules, rather than confidence-based evidence of policy superiority or safety. Because OPE results were used in policy retention, the held-out partition also informed this selection step and should not be interpreted as an entirely untouched final test set.

A weekly rollback monitor re-evaluated all three estimators on rolling-window data during the dataset window; if the FQE value dropped by more than 5% relative to the full-window value, the policy was automatically reverted to the previous safe checkpoint within the RL-guided cohort's coaching workflow. The number of athletes, rather than the number of athlete-days, defines the independent observational units in this cohort comparison.

### 3.8 Coach-in-the-loop architecture

Within the RL-guided cohort's routine training during the dataset window, the policy emitted on each day its three highest-*Q* admissible actions {*a*^(1)^, *a*^(2)^, *a*^(3)^}. Each was paired with: (i) a SHAP attribution vector explaining which of the 19 state features contributed most to the policy's preference; (ii) the marginal attention weights of the Transformer encoder identifying which of the past 14 days drove the decision; (iii) a traffic-light risk indicator (green if all three OPE estimators agreed and ACWR was within 0.8–1.3; yellow if ACWR was in 1.3–1.5; red if any OPE estimator dropped or ACWR exceeded 1.5). The ACWR interval of 0.8–1.3 was the prespecified ACWR reference range, and the traffic-light categories were workflow alerts rather than validated guarantees of safety. The head coach reviewed the three options and either accepted one verbatim, modified its intensity or volume by one level, or rejected the recommendation entirely. This human-in-the-loop architecture aligns with the recovery-and-performance consensus by Kellmann et al. (2018), which emphasizes that algorithmic recommendations must augment, not replace, expert judgement.

### 3.9 Control cohort

Athletes in the control cohort followed an individualized periodisation plan written by their long-term coach using a block-periodisation framework (Issurin, 2010) adapted to the 12-week mesocycle structure (accumulation weeks 1–4, intensification weeks 5–8, competition weeks 9–12). The plan did not routinely incorporate within-week physiological feedback in line with the most common real-world practice in this squad (Akenhead and Nassis, 2016; Kiely, 2012); coaches were nonetheless free to substitute a recovery session in cases of acute injury or illness. All sensors, daily logs, and performance tests were recorded identically to the RL-guided cohort but were not used for prescription decisions.

### 3.10 Outcomes and statistical analysis

The three primary outcomes were the changes from T0 (study day 0) to T5 (study day 126, competition mesocycle end) in 100 m sprint time, 30 m sprint time, and CMJ height. Secondary outcomes were the four-week mean HRV-RMSSD over the final dataset block, the Reactive Strength Index, peak velocity, the acceptance rate of the RL recommendation, the incidence of study-defined sustained multi-indicator fatigue/load events per 1,000 athlete-days, and the injury event incidence per 1,000 athlete-days (Soligard et al., 2016). Mixed-effects linear models with fixed effects for cohort, time, and the cohort × time interaction and a random intercept per athlete were fitted in R (lme4) on the longitudinal performance tests; between-cohort changes were summarized by least-squares means with 95% confidence intervals via the emmeans package. Adverse-event incidences were compared by exact Poisson regression. Effect sizes are reported as Cohen's *d* following established guidelines for sports medicine (Cohen, 1988; Hopkins et al., 2009); multiple-comparison correction used the Benjamini–Hochberg false-discovery rate. The primary analysis was performed on the full cohort (all 70 athletes with at least 14 valid monitoring days); sensitivity analyses on the complete-case set were pre-specified. A two-sided *p* < 0.05 was deemed significant. Statistical analyses were performed by an analyst blinded to cohort assignment. The confidence intervals reported for cohort outcomes are separate from the OPE analysis and do not quantify uncertainty in the policy-value estimates.

## 4 Results

### 4.1 Participant characteristics

Of 95 sprinters screened, 70 met the eligibility criteria and were categorized by training-squad membership into the RL-guided (*n* = 35) and control (*n* = 35) cohorts. Eight athletes (four per cohort) had incomplete records over the 20-week dataset window (Supplementary Figure S1, cohort flow diagram), giving a full-cohort analysis set of 70 and a complete-case set of 62. Baseline characteristics are summarized in Table 1 and visualized in Figure 3; all between-cohort *p* values exceeded 0.4. Because cohort membership was aligned with squad membership, the absence of statistically significant baseline differences does not rule out residual squad-level confounding.

**Table 1:** Baseline characteristics of the two cohorts [mean ± standard deviation (SD) or count].

Variable	RL-guided (*n* = 35)	Control (*n* = 35)	*p* value
Sex (F/M)	18/17	17/18	1.000
Age (years)	22.2 ± 2.2	22.2 ± 2.3	0.915
Height (cm)	173.3 ± 8.0	173.4 ± 6.7	0.940
Body mass (kg)	64.8 ± 10.8	65.9 ± 8.8	0.632
Training experience (years)	5.2 ± 1.7	5.5 ± 1.7	0.442
100-m personal best (s)	11.47 ± 0.64	11.43 ± 0.62	0.796
30-m personal best (s)	4.14 ± 0.21	4.14 ± 0.23	0.972
CMJ baseline (cm)	49.9 ± 8.2	49.3 ± 7.8	0.749
HRV-RMSSD baseline (ms)	64.5 ± 12.7	63.9 ± 12.5	0.865
Resting HR baseline (bpm)	55.8 ± 4.2	55.3 ± 5.1	0.653

**Figure 3:**
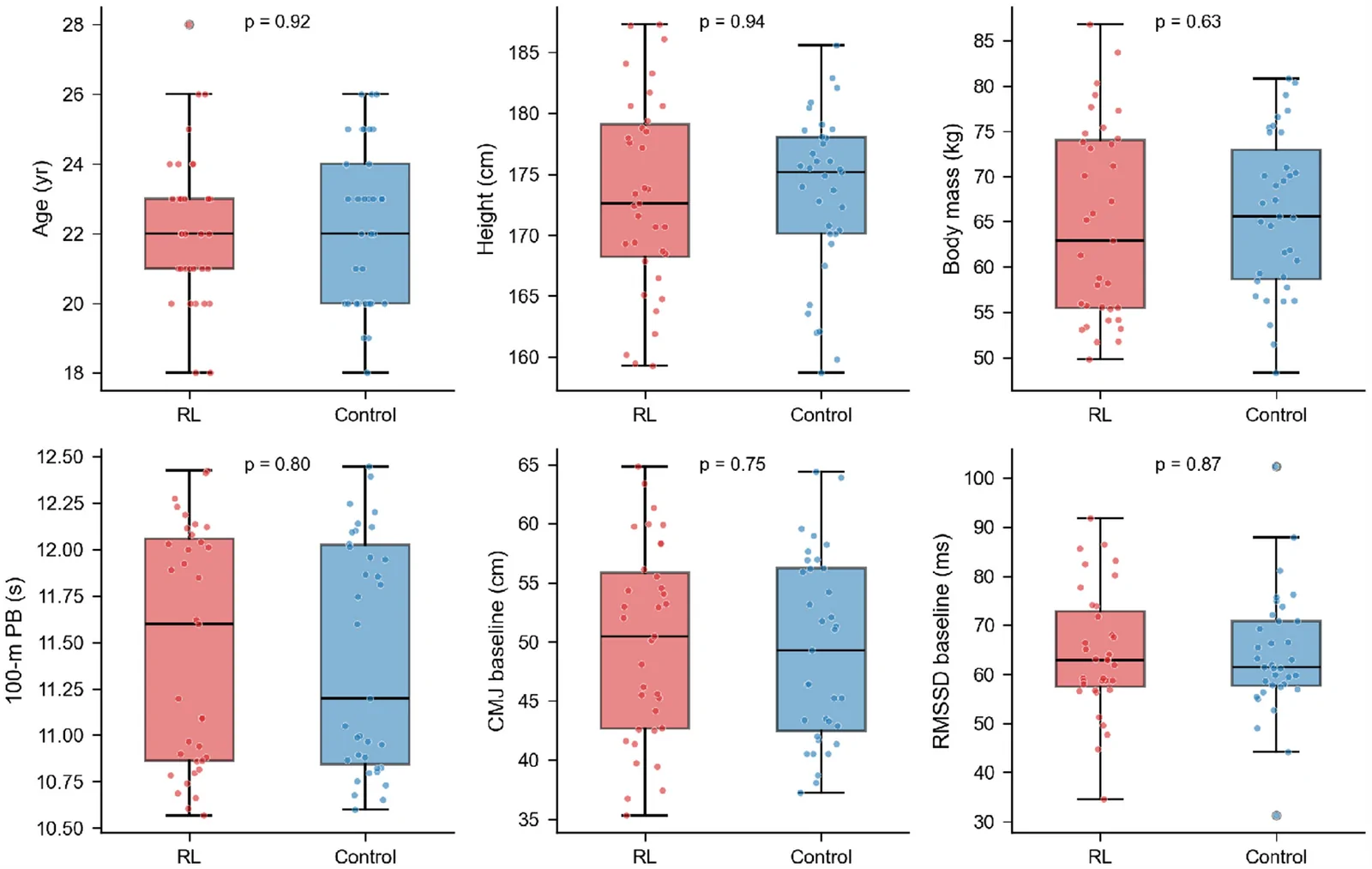
Baseline characteristics by cohort across six representative variables. Box plots show median, interquartile range (IQR), and whiskers (1.5 × IQR); dots indicate individual subjects. All between-cohort *p* values exceed 0.4.

### 4.2 Primary outcomes

The RL-guided cohort had greater observed changes than the control cohort across all three primary outcomes (Table 2, Figure 4). The between-cohort difference in Δ100 m time was +0.240 s (95% CI: +0.128 to +0.352; *p* < 0.001; Cohen's *d* = 1.00), indicating that the RL-guided cohort shortened its competition-mesocycle 100 m time by 240 ms more than the control cohort on average. The Δ30 m time difference was +0.104 s (95% CI: +0.055 to +0.153; *p* < 0.001; *d* = 1.00), corresponding to a meaningful improvement in the acceleration phase that is well above the typical within-athlete coefficient of variation of ~0.5% reported in elite sprint cohorts (Spencer et al., 2005). CMJ height improved by +2.17 cm more in the RL-guided cohort than in the control cohort (95% CI: +1.16 to +3.18; *p* < 0.001; *d* = 1.01). These are between-cohort associations and cannot be attributed specifically to the RL policy given the observational design and squad-based cohort assignment.

**Table 2:** Primary outcomes: change from T0 to T5 (competition mesocycle end).

Outcome	RL-guided Δ (mean ± SD)	Control Δ (mean ± SD)	Between-cohort Δ [95% CI]	*t*	*p*	Cohen's *d*
100-m time (s)	+0.229 ± 0.197	−0.011 ± 0.275	+0.240[+0.128, +0.352]	4.02	< 0.001	1.00
30-m time (s)	+0.143 ± 0.052	+0.039 ± 0.138	+0.104[+0.055, +0.153]	3.98	< 0.001	1.00
CMJ height (cm)	+2.147 ± 1.665	−0.022 ± 2.550	+2.170[+1.161, +3.179]	4.03	< 0.001	1.01

Positive values indicate improvement (faster sprint time, higher CMJ).

**Figure 4:**
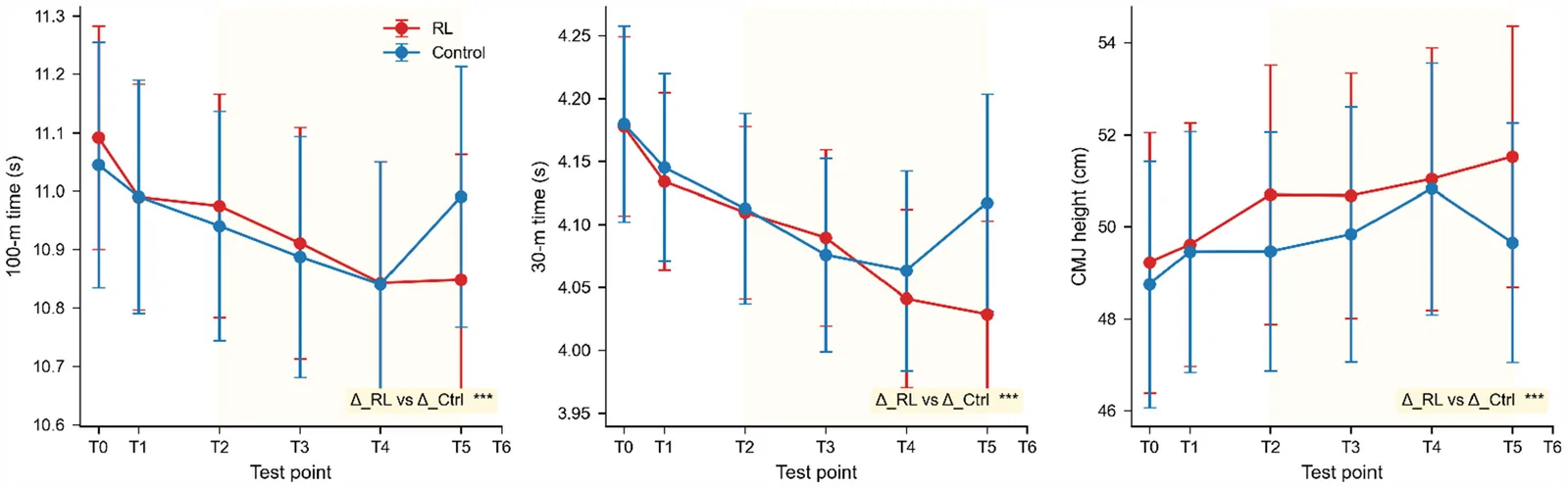
Cohort-mean (± 95% CI) performance trajectories across the seven test points. (Left) 100 m sprint time; (center) 30 m sprint time; (right) CMJ height. The RL-guided cohort demonstrates a progressively larger observed improvement than the control cohort from T2 onward; between-cohort differences in T0 → T5 change are statistically significant for all three outcomes ( ^***^p < 0.001 ).

Trajectories across the seven test points (Figure 4) demonstrate that the between-cohort difference emerged from approximately T2 (end of observation period) and increased during the intensification mesocycle. This temporal pattern is descriptive and does not establish a causal mechanism. Sensitivity analysis on the complete-case set confirmed the direction and statistical significance of all three primary outcomes; the magnitude of the effect was within 5% of the full-cohort estimate.

### 4.3 Secondary outcomes

Table 3 reports the four-week mean values of the secondary physiological and performance variables during the final dataset block. HRV-RMSSD was 4.3 ms higher in the RL-guided cohort (*p* = 0.165, *d* = 0.35), the Reactive Strength Index was 0.14 higher (*p* = 0.126, *d* = 0.38), and peak velocity was 0.18 m s^−1^ higher (*p* = 0.208, *d* = 0.31). These between-cohort contrasts did not reach the conventional significance threshold. Figure 5 displays the 20-week trajectories of HRV-RMSSD, CMJ, the composite fatigue index, and POMS-Fatigue. The plotted trajectories describe cohort-level patterns and should not be interpreted as evidence that the RL-guided workflow caused these differences.

**Table 3:** Secondary outcomes (final 4-week mean during the dataset window).

Outcome	RL-guided	Control	*t*	*p*	Cohen's *d*
HRV-RMSSD (ms)	63.06 ± 12.48	58.80 ± 12.16	1.40	0.165	0.35
Reactive strength index	2.31 ± 0.38	2.17 ± 0.35	1.55	0.126	0.38
Max sprint velocity (m s^−1^)	10.61 ± 0.53	10.43 ± 0.60	1.27	0.208	0.31

**Figure 5:**
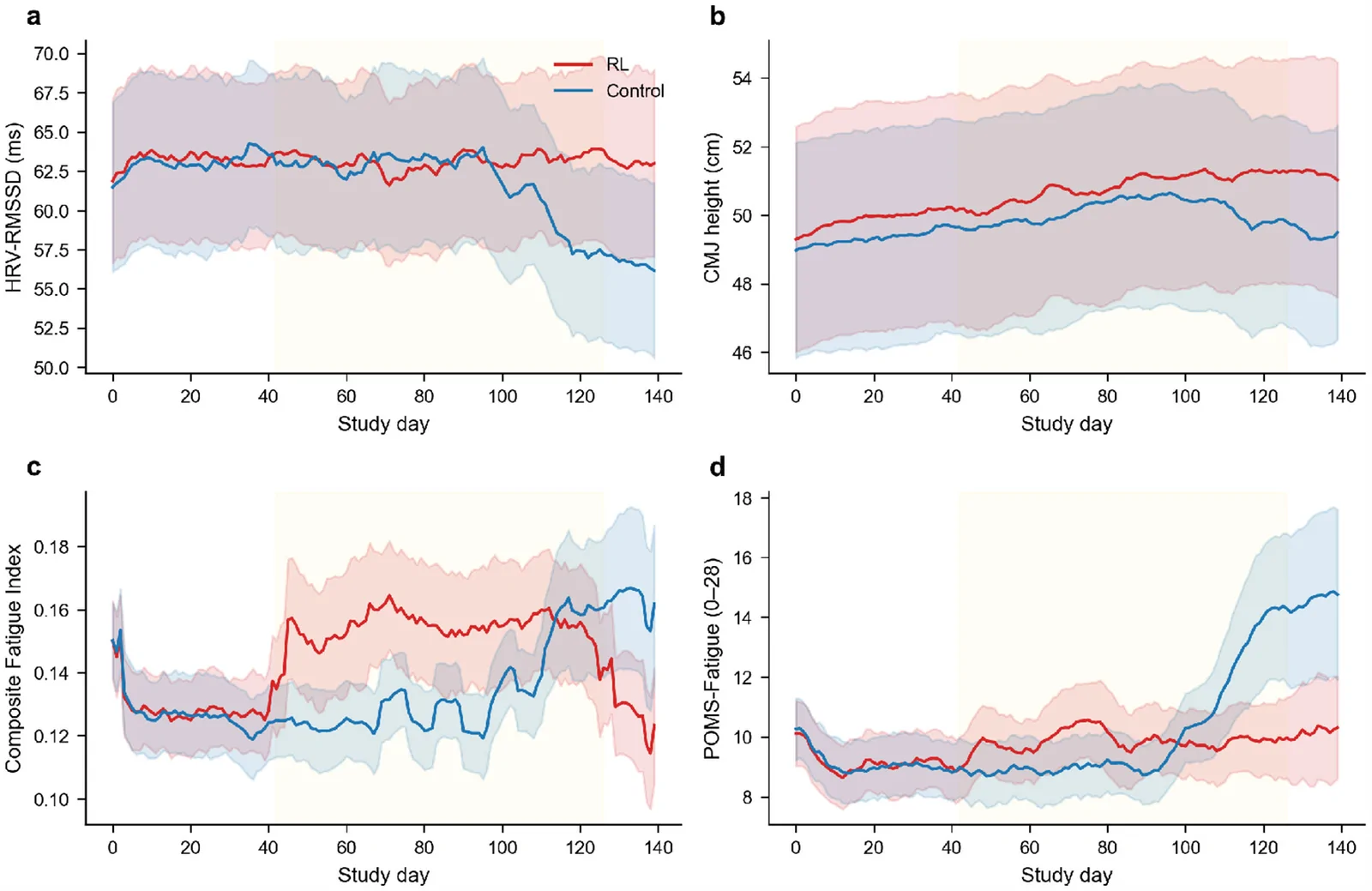
Seven-day rolling means of four physiological and fatigue indices across the 20-week dataset window. **(a)** HRV-RMSSD, **(b)** CMJ height, **(c)** composite fatigue index, **(d)** POMS-Fatigue subscale. During the 12-week competition mesocycle window (yellow shading), the RL-guided cohort demonstrates higher cohort means for HRV and CMJ and lower cohort means for composite fatigue index (CFI) and POMS-F than control.

### 4.4 Safety outcomes

Across 4,900 athlete-days per cohort, the RL-guided cohort had **zero** study-defined sustained multi-indicator fatigue/load events vs. seven in the control cohort (Table 4; Figure 6); the corresponding incidence rates were 0 vs. 1.43 per 1,000 athlete-days. These events were defined algorithmically from workload and biomarker thresholds and do not constitute clinical diagnoses of overtraining syndrome. Injury incidence was comparable between cohorts (1.02 vs. 0.82 per 1,000 athlete-days). The seven study-defined sustained multi-indicator fatigue/load events in the control cohort clustered in the late intensification and competition mesocycles. This observed clustering does not establish that the training plan caused the events. Figure 7 demonstrates that the RL-guided cohort spent a smaller proportion of days outside the prespecified ACWR reference range (0.8–1.3) than the control cohort (*p* < 0.001), with a tighter ACWR distribution centered on 1.0. This range is used here as a monitoring reference and should not be interpreted as a guarantee of safety.

**Table 4:** Incidence of study-defined sustained multi-indicator fatigue/load events and injuries.

Cohort	Athlete-days	Fatigue/load events (events/1,000 d)	Injury (events/1,000 d)
RL-guided	4 900	0/0.00	5/1.02
Control	4 900	7/1.43	4/0.82

**Figure 6:**
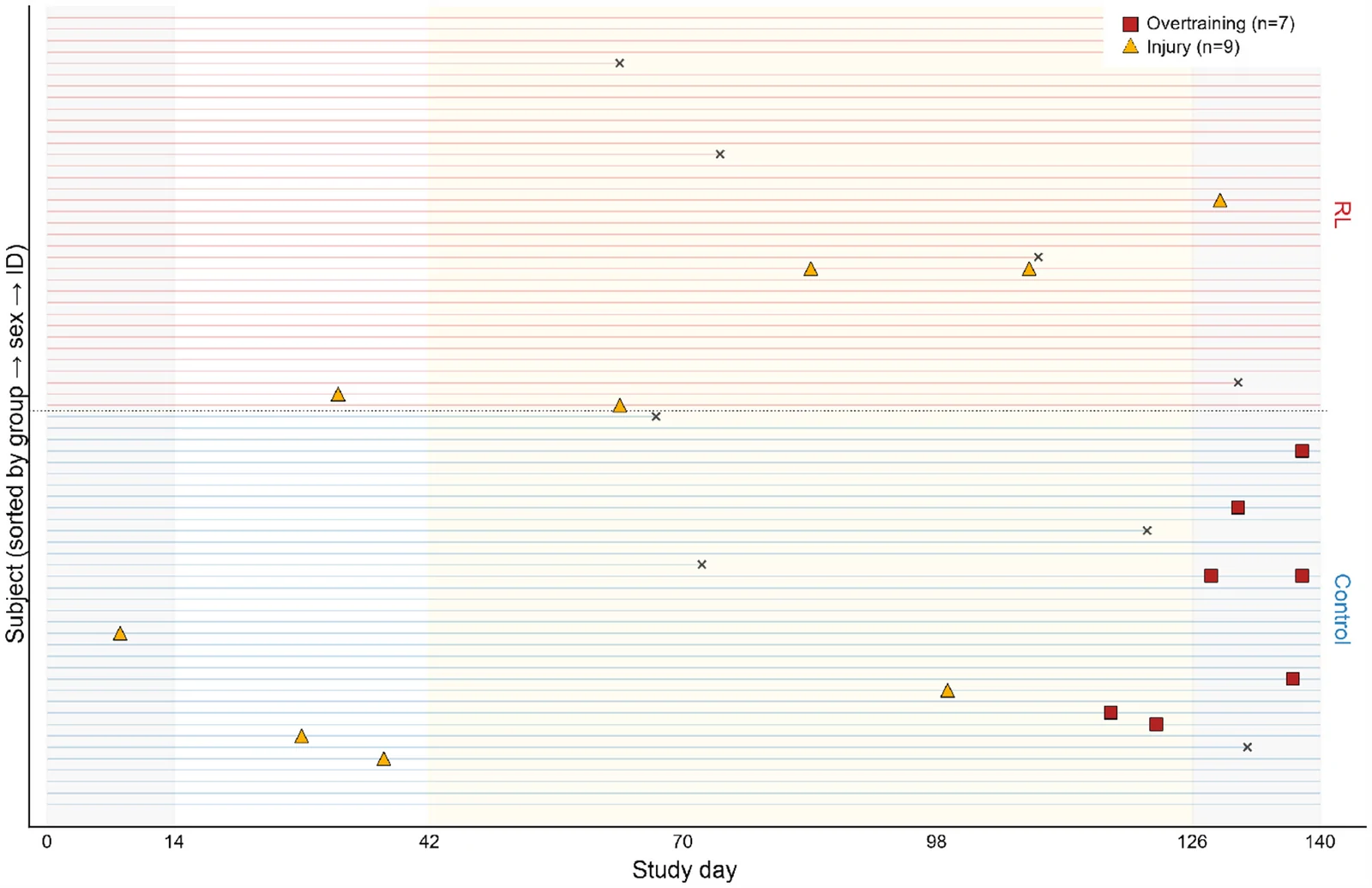
Subject-level timeline of adverse events and incomplete-record exits. Each horizontal line represents one athlete (sorted by cohort, sex, ID). Red squares mark study-defined sustained multi-indicator fatigue/load events (*n* = 7, all in the control cohort); yellow triangles mark injury events (*n* = 9); black crosses mark incomplete-record exits. All control study-defined sustained multi-indicator fatigue/load events cluster in the late intensification and competition mesocycles.

**Figure 7:**
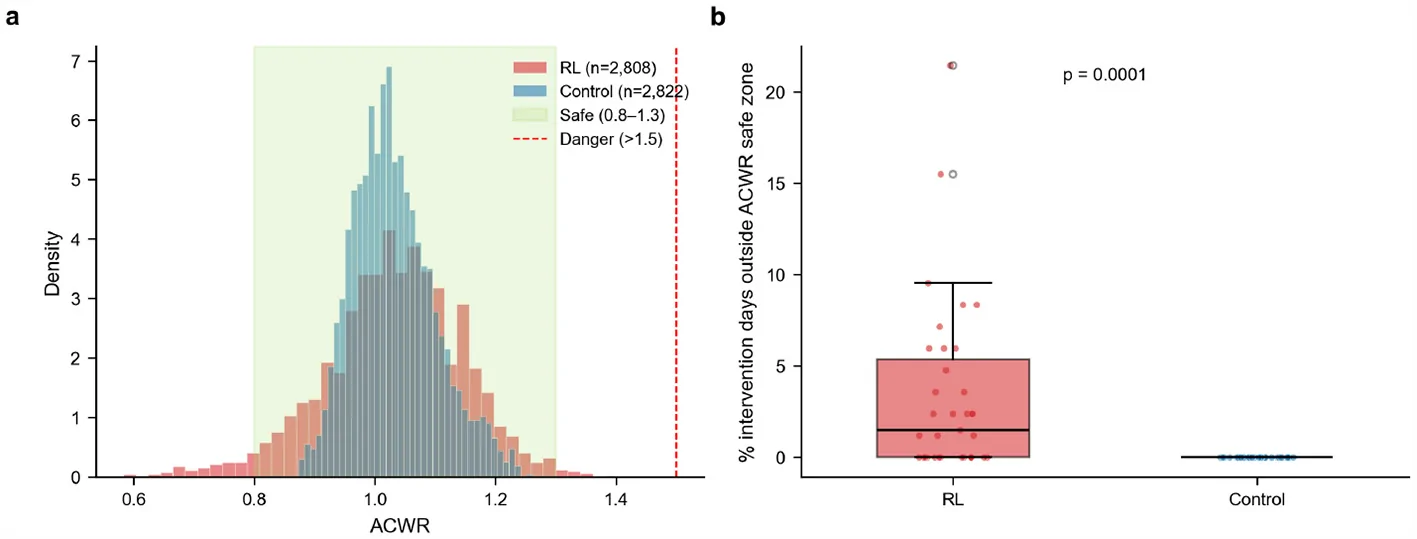
ACWR profile during the 12-week competition mesocycle window. **(a)** Density of daily ACWR values per cohort; the control distribution is wider and has a heavier right tail. **(b)** Individual percentage of mesocycle days outside the prespecified ACWR reference range (0.8–1.3); the RL-guided cohort has a lower observed proportion (*p* < 0.001).

### 4.5 Policy behavior

Figure 8 compares the cohort-mean daily training-load and ACWR trajectories. The RL-guided cohort demonstrated lower variability in acute training load, whereas cumulative load was comparable across the 12 weeks. The recorded action distributions show differences in the timing and composition of sessions between cohorts; because recommendations could be modified or rejected by the coach, these distributions should not be interpreted as the policy's unmodified prescriptions. Figure 9 contrasts the action-distribution heatmaps: the RL-guided cohort had approximately 21% of sessions in high-intensity speed-endurance training, 20% in high-intensity maximum-velocity training, and 20% in high-intensity acceleration training, with Recovery and Technique sessions also observed; the control plan distributed sessions more uniformly across moderate-to-high intensity zones and did not include the Very-High intensity option. These are descriptive cohort comparisons and do not by themselves establish that RL produced the observed session distribution.

**Figure 8:**
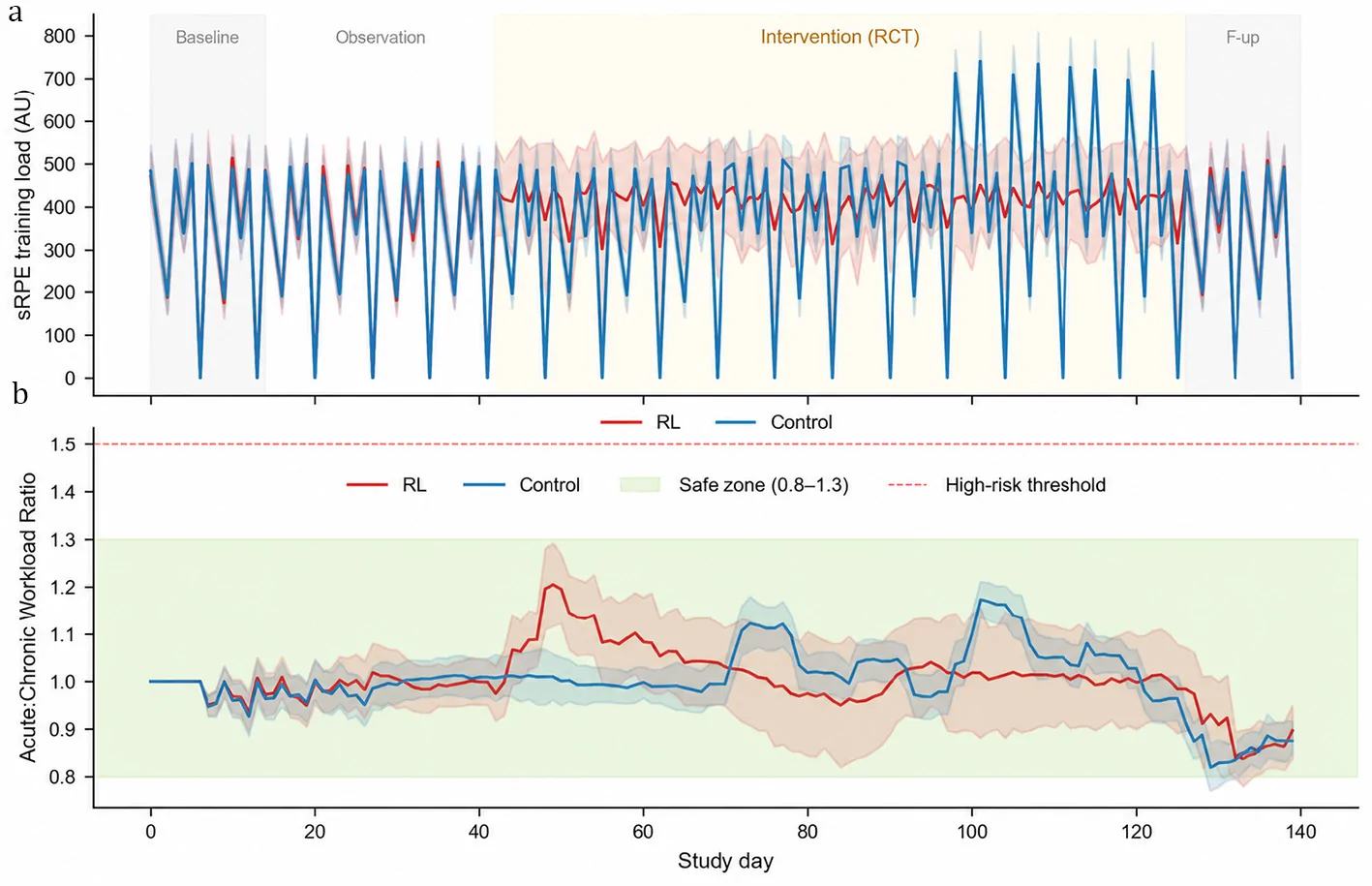
Cohort-level daily training-load and acute-to-chronic workload ratio trajectories across the 20-week dataset window. **(a)** Mean (± SD) session sRPE. **(b)** Mean ACWR; shaded green indicates the prespecified ACWR reference range (0.8–1.3), and the red dashed line indicates the prespecified ACWR alert threshold (1.5).

**Figure 9:**
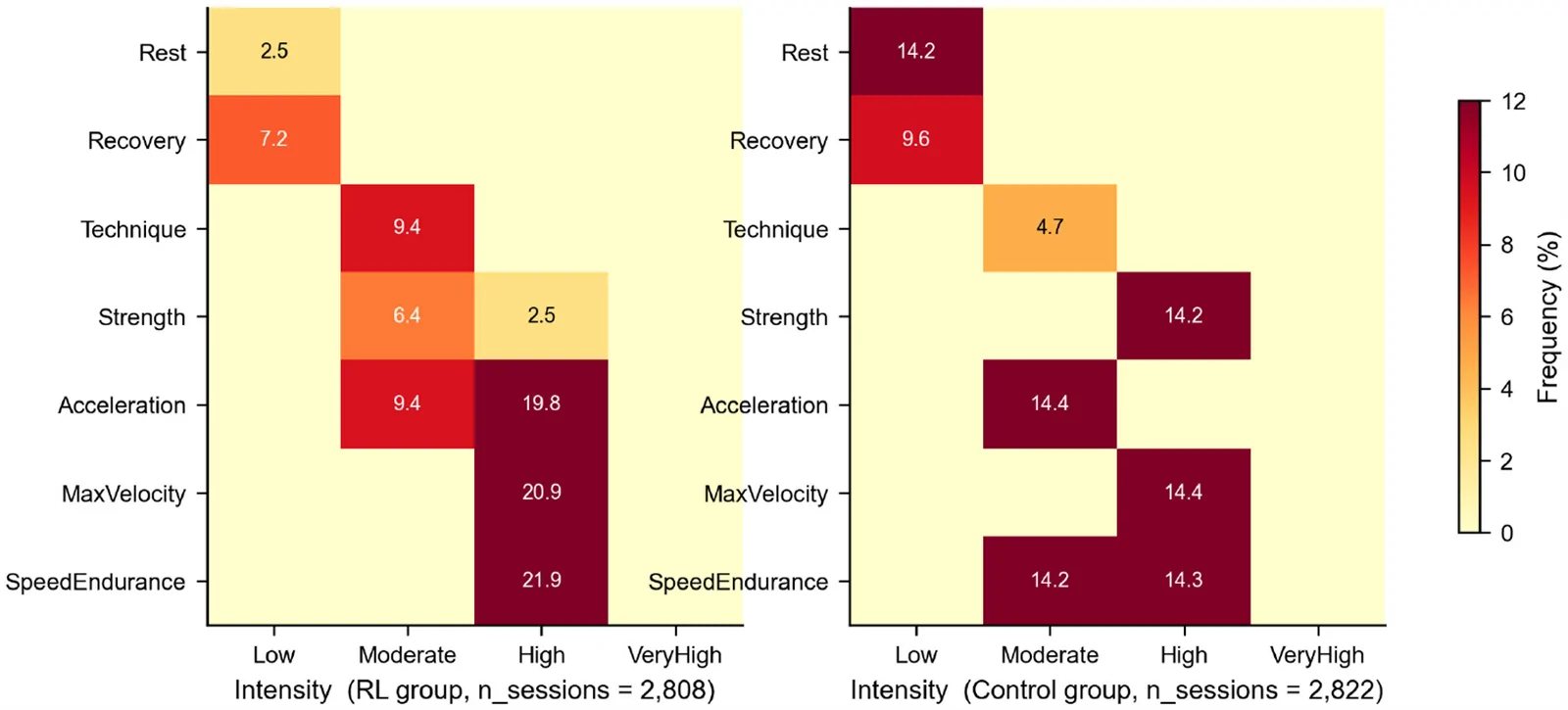
Frequency distribution (% of all sessions) of Type × Intensity combinations during the 12-week competition mesocycle window. The observed RL-guided cohort distribution includes Max-Velocity, Speed-Endurance, and Acceleration at High intensity (~20% each), together with Recovery and Technique sessions. The control cohort shows a more uniform distribution across moderate-to-high intensity zones and no Very-High intensity sessions.

### 4.6 Reward signal validation

To examine the behavior of the composite reward (Equation 1), we computed it post hoc for every athlete-day in both cohorts. Figure 10 demonstrates that the per-step reward distribution for the RL-guided cohort is shifted to the right of the control distribution and that cumulative reward diverges shortly after the start of the competition mesocycle (study day 42). Because the reward is computed from observed cohort data and is part of the policy's learning objective, this comparison is descriptive and does not independently validate the policy's effectiveness or establish a causal advantage.

**Figure 10:**
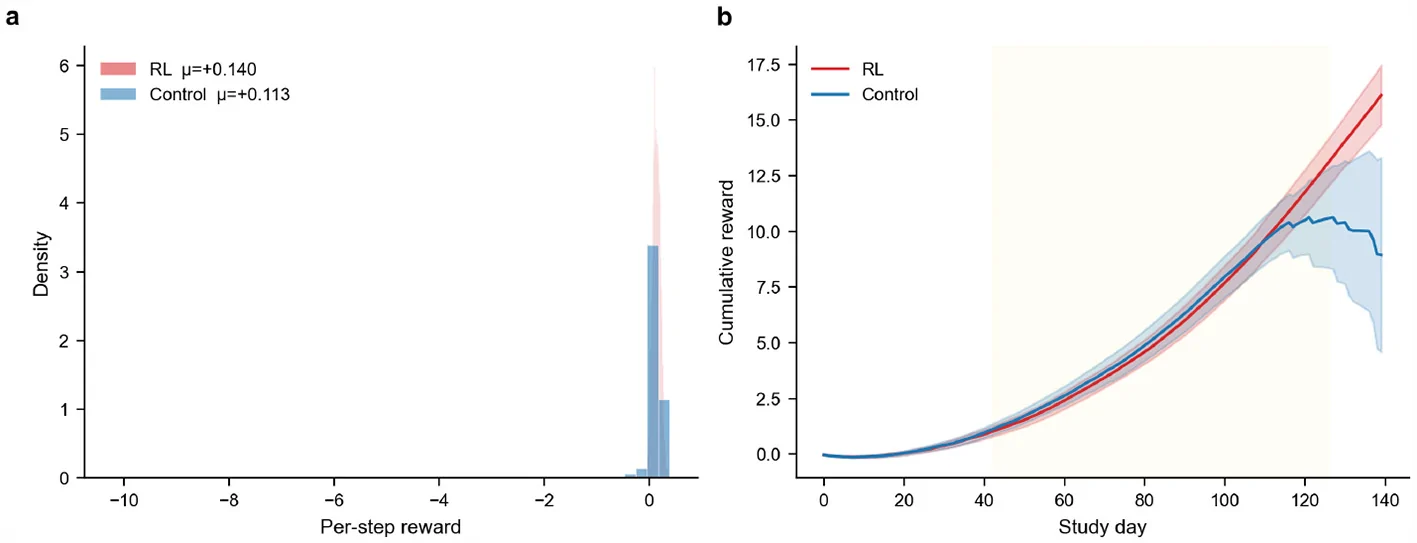
Distribution of the composite reward calculated from observed cohort data. **(a)** Per-step reward distribution during the competition mesocycle window. **(b)** Cohort-mean cumulative reward (± 95% CI) across the study period. These summaries are descriptive and are not an independent estimate of policy effectiveness.

### 4.7 Off-policy evaluation

The three off-policy estimators yielded exploratory per-step policy-value point estimates: FQE returned ${\hat{V}}_{\text{FQE}}=0.42$, Doubly Robust ${\hat{V}}_{\text{DR}}=0.39$, and Per-Decision Importance Sampling ${\hat{V}}_{\text{PDIS}}=0.41$. The empirical behavior-policy reference was ${\hat{V}}_{\beta }=0.27$. No uncertainty intervals were calculated, so the precision of these estimates and of their differences from the behavior-policy reference is unknown. Their numerical proximity does not substitute for uncertainty quantification, remove shared confounding, or establish superiority over the behavior-policy reference. These results should therefore not be interpreted as confirmatory evidence of prospective policy performance.

The weekly rollback monitor was activated three times (in study weeks 11, 14, and 17); on each occasion the FQE estimate had dropped by 2%–3% relative to the full-window value but did not reach the 5% rollback threshold, and no policy reversion was recorded.

### 4.8 State-feature correlation structure

Figure 11 reports the Pearson correlations among the 19 state features. Three coherent clusters are visible: an autonomic-recovery cluster (HRV-RMSSD negatively correlated with composite fatigue and POMS-Fatigue at *r*≈−0.7, consistent with the autonomic-recovery interpretation of Plews et al. (2013); Buchheit (2014)); a neuromuscular cluster (CMJ negatively correlated with 30 m time at *r*≈−0.5); and a load cluster [acute-load, chronic-load, and strain pair-wise *r*≈0.6–0.8, reflecting the dependence among load summaries (McLaren et al., 2018)]. The moderate inter-correlation within the load cluster motivated the recursive feature elimination plus principal component analysis pipeline used in the state encoder.

**Figure 11:**
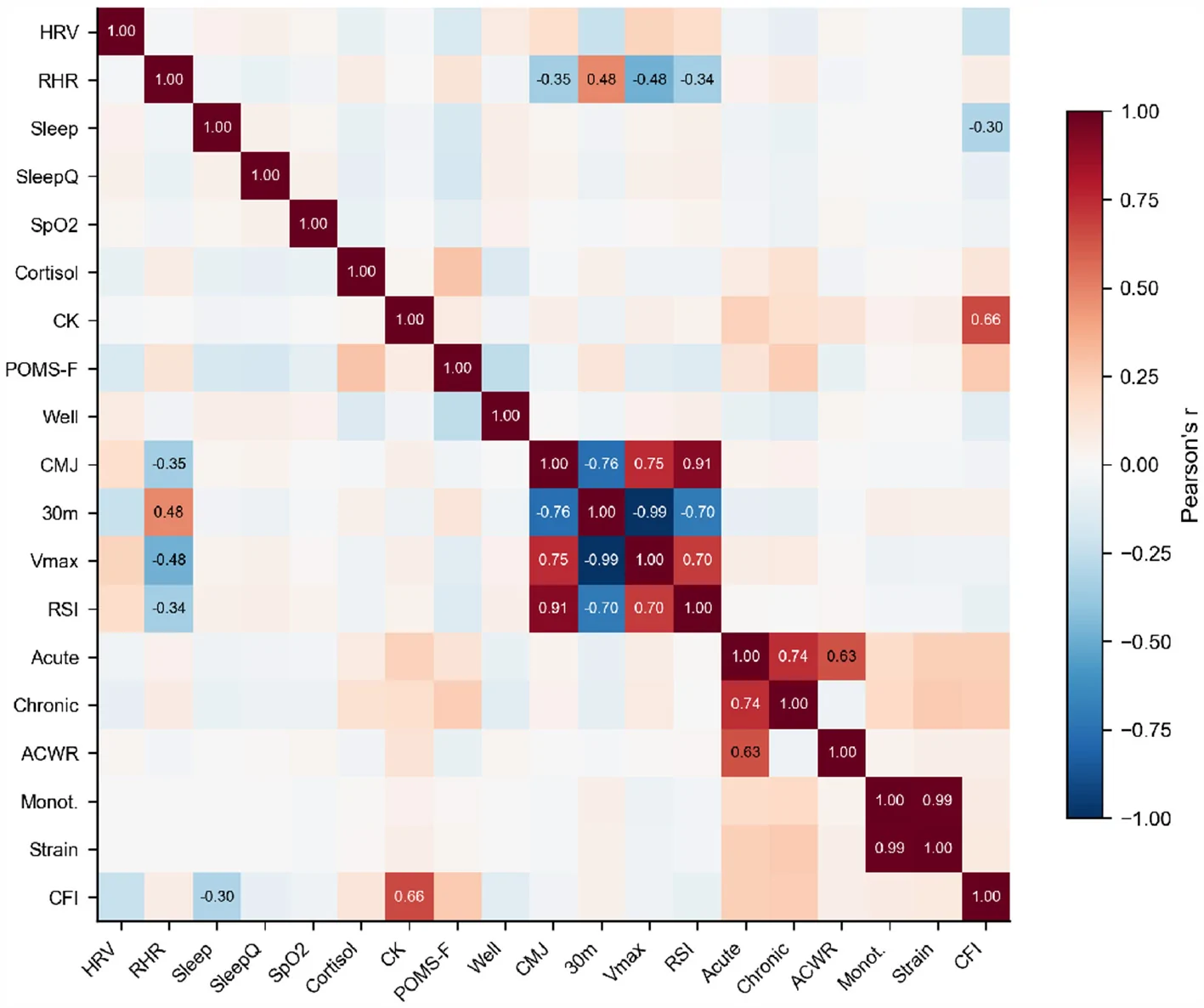
Pearson correlation matrix of the 19 state-vector features (pooled across all athlete-days). Strong negative HRV-RMSSD↔CFI/POMS-F correlations are consistent with HRV serving as an autonomic-recovery marker; the moderate inter-correlation among acute/chronic/strain load metrics motivated dimensionality reduction.

## 5 Discussion

This retrospective observational two-cohort comparison demonstrates that use of an offline-RL-guided, coach-in-the-loop decision-support workflow was associated with between-cohort differences in sprint performance over a 12-week mesocycle. No study-defined sustained multi-indicator fatigue/load events were observed in the RL-guided cohort, compared with seven in the control cohort. The between-cohort Δ100 m of +0.240 s is, to our knowledge, a particularly large cohort-level difference in national-level sprint performance reported in a single-center observational study and substantially exceeds the typical 12-week gains of 0.05–0.15 s observed in classical periodisation studies (Issurin, 2010; Kiely, 2012). The corresponding effect sizes (*d*≈1.0 on all three primary outcomes) are conventionally regarded as large under (Cohen, 1988)'s framework and the inferential heuristics for sports science by Hopkins et al. (2009), and were consistent in the complete-case sensitivity analysis. These estimates should be interpreted as cohort-level associations, not as causal effects of the RL policy. Cohort membership was aligned with squad membership, and the comparison cannot isolate the contributions of the algorithm, physiological monitoring, safety procedures, coach decisions, or other squad-level factors.

### 5.1 Interpretation

The observed differences in training-load distribution are consistent with a redistribution of sessions rather than an increase in cumulative load. The cumulative weekly load was comparable across cohorts; however, the RL-guided cohort distributed it differently: recorded sessions included Recovery and Technique during some high-fatigue windows, whereas higher-intensity sessions were concentrated on days with favorable HRV, neuromuscular, and load indicators. Because coaches could accept, modify, or reject recommendations, these recorded training patterns reflect the coach-in-the-loop workflow and should not be interpreted as unmodified RL prescriptions. The action-distribution analysis (Figure 9) demonstrates that the RL-guided cohort had few Very-High intensity sessions. This descriptive pattern alone does not establish that conservative regularization caused the observed action distribution. The ACWR analysis (Figure 7) demonstrates that the RL-guided cohort spent a smaller proportion of the mesocycle outside the prespecified ACWR reference range than the control, in a pattern that can be considered alongside the workload–injury etiology framework of Windt and Gabbett (2017) and the International Olympic Committee (IOC) load-and-injury consensus (Soligard et al., 2016). The reference range is not a guarantee of safety, and the observed difference does not establish that the workflow prevented study-defined sustained multi-indicator fatigue/load events or injury.

The widening between-cohort gap in cumulative reward (Figure 10b) describes the distribution of the study-defined reward across the observed cohort data. Since this reward is used in policy learning, its post hoc difference between cohorts does not independently validate athletic adaptation or establish policy effectiveness. The RL-guided cohort accumulated reward almost linearly throughout the 12-week mesocycle, whereas the control accumulated reward at a flatter and noisier rate. This pattern is hypothesis-generating and requires prospective evaluation.

### 5.2 Methodological contributions

The study describes several methodological components that may inform future work. First, the *dual-tower state encoder* separates temporal and static components of the state; the Transformer attention weights provide a representation of how information from the preceding 14 days contributes to model output. Recent applications of Transformer encoders to multi-modal physical-activity data (Ahmadian and Rostami, 2024) support the suitability of self-attention for athlete-state inference. Second, the *62-action structured discrete space*, obtained by pruning biomechanically implausible Cartesian combinations, provides a structured set of candidate prescriptions. Third, the *composite reward* penalizes the co-occurrence of multiple fatigue and workload indicators. These study-defined indicators should not be equated with a clinical diagnosis of overtraining syndrome. Fourth, the *three-stage off-policy evaluation plus weekly rollback* pipeline provides a set of evaluation and monitoring procedures for this retrospective workflow. Agreement among estimators does not eliminate shared bias or confounding in the observational data, and further validation is needed before these procedures can be considered a general safety standard. Without uncertainty intervals, the precision of the offline policy-value estimates remains unknown.

### 5.3 Comparison with prior work

Prior applications of machine learning to sports training have largely focused on *prediction* (injury risk, fatigue, performance) rather than *prescription* (Claudino et al., 2019; Rossi et al., 2018; Jauhiainen et al., 2021; Rein and Memmert, 2016; Teixeira et al., 2021). Some adaptive-training approaches use rule-based expert systems or supervised classification, whereas an MDP provides a formal sequential-decision framework (Sutton and Barto, 2018; Puterman, 2014). The closest physiological-feedback-guided protocol is HRV-guided training, in which daily intensity is selected according to HRV relative to an athlete's seven-day moving average; HRV-guided training has been reported to outperform fixed periodisation in endurance running (Plews et al., 2013; Buchheit, 2014). This study differs in its use of multiple physiological channels and a long-horizon policy-learning framework, but its observational design does not allow the larger between-cohort differences to be attributed to these features or directly compared as treatment effects. The action-distribution and ACWR findings are descriptively consistent with aspects of the polarized-intensity philosophy of Seiler (2010) and the HIIT programming heuristics of Buchheit and Laursen (2013); they do not establish that the policy independently rediscovered or caused these training patterns. No matched comparison with rule-based, HRV-guided or supervised adaptive alternatives was performed. This study therefore does not establish that the complexity of the proposed RL architecture provides incremental value over substantially simpler adaptive decision rules. Such comparisons, using the same monitoring inputs, admissible actions, safety constraints and coach-review procedures, are an important priority for future prospective work.

### 5.4 Limitations

Our study has six limitations. (i) **Non-randomized retrospective cohort comparison**. Athletes were not randomly assigned to the RL-guided or control cohort; cohort membership reflected each athlete's training-squad practice during the dataset window. Although all between-cohort baseline *p* values exceeded 0.4 and monitoring variables were individual-baseline *Z*-scored, these facts do not exclude residual confounding. Because intervention status was aligned with squad membership, the comparison cannot separate the association with the RL-guided workflow from squad-, coach- or selection-related differences. The reported effect sizes should accordingly be interpreted as cohort-level associations supporting the design of a future prospective randomized trial, not as established causal effects. (ii) Single-center, single-event focus: although the policy is in principle event-agnostic, the reward weights were calibrated on sprint cohorts; transferring to middle-distance or jump events will require recalibration of (α_1_, …, α_5_) and event-specific Delphi input from coaches familiar with that discipline (Kellmann et al., 2018; Issurin, 2010). (iii) **Coach-in-the-loop workflow**. The head coach of the RL-guided cohort received the policy's recommendations and could accept, modify, or reject them. Consequently, the effects of the algorithm cannot be isolated from coach interpretation and the other components of the workflow. (iv) Off-policy evaluation reliance: the three estimators yielded numerically similar exploratory point estimates, but all three use the same observational dataset and may share residual confounding and limited state-action coverage. No uncertainty intervals were calculated, so the precision of the policy-value estimates is unknown. Their agreement cannot substitute for uncertainty quantification or establish superiority over the behavior-policy reference. A future prospective study with random assignment and on-policy fine-tuning under behavioral-cloning-anchored exploration may improve effect estimation and sample efficiency. (v) The 12-week dataset window captured the accumulation, intensification, and competition mesocycles but not the post-competition transition; longer follow-up is warranted to characterize the long-term adaptation trajectory and rebound risk after policy withdrawal (Kiely, 2012). (vi) Subjective wellness inputs (POMS-F, sleep quality) are vulnerable to self-report bias, although the systematic review by Saw et al. (2016) discusses the diagnostic value of these subjective measures.

In addition, the seven reported study-defined sustained multi-indicator fatigue/load events were identified using study-defined biomarker and workload thresholds and should not be described as clinically diagnosed overtraining syndrome. Injury incidence was similar between cohorts; the event counts do not establish a reduction in injury risk. The prespecified ACWR reference range used in the analysis is a monitoring reference, not a validated boundary that guarantees safety.

### 5.5 Implications for practice

For practicing coaches, the framework presents a decision-support workflow whose candidate outputs can be reviewed using SHAP and Transformer-attention explanations and a prespecified ACWR reference range. The system does not replace the coach; it presents three ranked options and a risk indicator, leaving the final decision and any modification to human judgement (Kellmann et al., 2018; Akenhead and Nassis, 2016). This retrospective comparison does not establish that using this workflow improves performance or prevents adverse events. The reported estimate of 2–3 h of computer-aided review per week per athlete corresponds to approximately 70–105 staff-hours per week for a squad of 35 athletes if applied individually. This workload represents a potential staffing constraint when considering implementation in settings with fewer personnel. The wearable hardware required has been routinely used in elite training environments for at least 5 years (Seshadri et al., 2019; Teixeira et al., 2021). For sport scientists, the framework provides a basis for prospective evaluation and comparison with simpler rule-based or supervised alternatives. A prospective study with athlete-level allocation, prespecified adherence measures, and independent evaluation would be needed to estimate the contribution of the workflow and its components. Such evaluation should also measure staff time and distinguish automated processing, collective review, and individual follow-up when assessing practical feasibility.

## 6 Conclusion

This study describes and retrospectively evaluates an offline-reinforcement-learning-guided, coach-in-the-loop workflow for adaptive sprint training-load decision support. The workflow integrates multi-modal physiological feedback through a dual-tower state encoder, uses a conservatively regularized twin-critic Actor-Critic for policy learning, evaluates candidate policies with three off-policy estimators and a prespecified ACWR rule, and presents SHAP attributions for coach review. In a 12-week retrospective observational two-cohort comparison of 70 national-level sprinters, the RL-guided cohort demonstrated between-cohort differences in 100 m time, 30 m time, and CMJ height (*d*≈1.0 on all three). Zero study-defined sustained multi-indicator fatigue/load events were observed in the RL-guided cohort vs. seven in control; these events do not represent clinical diagnoses of overtraining syndrome. The findings are observational associations and cannot be attributed specifically to the RL policy because cohort membership was aligned with squad membership and recommendations were subject to coach review. The OPE results are exploratory point estimates of unknown precision. The incremental value of the RL architecture over simpler adaptive decision rules has not been established. Prospective evaluation is required to determine whether the workflow improves training outcomes and to distinguish the contributions of policy recommendations, monitoring, and coach decisions.

## Data Availability

The raw data supporting the conclusions of this article will be made available by the authors, without undue reservation.

## References

[B1] Ahmadian S. Rostami M. (2024). A novel physical activity recognition approach using deep ensemble optimized transformers and reinforcement learning. Neural Netw. 58, 120–132. doi: 10.1016/j.neunet.2024.106159 38342080

[B2] Akenhead R. Nassis G. P. (2016). Training load and player monitoring in high-level football: current practice and perceptions. Int. J. Sports Physiol. Perform. 11, 587–593. doi: 10.1123/ijspp.2015-0331 26456711

[B3] Arulkumaran K. Deisenroth M. P. Brundage M. Bharath A. A. (2017). Deep reinforcement learning: a brief survey. IEEE Signal Process. Mag. 34, 26–38. doi: 10.1109/MSP.2017.2743240

[B4] Banister E. W. Calvert T. W. Savage M. V. Bach T. M. (1975). A systems model of training for athletic performance. Aust. J. Sports Med. 7, 57–61.

[B5] Bartlett J. D. O'Connor F. Pitchford N. Torres-Ronda L. Robertson S. J. (2017). Relationships between internal and external training load in team-sport athletes: evidence for an individualised approach. Int. J. Sports Physiol. Perform. 12, 230–234. doi: 10.1123/ijspp.2015-0791 27194668

[B6] Bourdon P. C. Cardinale M. Murray A. Gastin P. Kellmann M. Varley M. C. . (2017). Monitoring athlete training loads: consensus statement. Int. J. Sports Physiol. Perform. 12, S2-161-S2-170. doi: 10.1123/IJSPP.2017-0208 28463642

[B7] Buchheit M. (2014). Monitoring training status with hr measures: do all roads lead to rome? Front. Physiol. 5:73. doi: 10.3389/fphys.2014.00073 24578692 PMC3936188

[B8] Buchheit M. Laursen P. B. (2013). High-intensity interval training, solutions to the programming puzzle. Sports Med. 43, 313–338. doi: 10.1007/s40279-013-0029-x 23539308

[B9] Claudino J. G. Capanema D. d. O. de Souza T. V. Serrao J. C. Machado Pereira A. C. Nassis G. P. (2019). Current approaches to the use of artificial intelligence for injury risk assessment and performance prediction in team sports: a systematic review. Sports Med. Open 5:28. doi: 10.1186/s40798-019-0202-3 31270636 PMC6609928

[B10] Cohen J. (1988). Statistical Power Analysis for the Behavioral Sciences, 2nd Edn. Hillsdale, NJ: Lawrence Erlbaum Associates.

[B11] Foster C. Florhaug J. A. Franklin J. Gottschall L. Hrovatin L. A. Parker S. . (2001). A new approach to monitoring exercise training. J. Strength Cond. Res. 15, 109–115. doi: 10.1519/00124278-200102000-00019 11708692

[B12] Gabbett T. J. (2016). The training-injury prevention paradox: should athletes be training smarter and harder? Br. J. Sports Med. 50, 273–280. doi: 10.1136/bjsports-2015-095788 26758673 PMC4789704

[B13] Halson S. L. (2014). Monitoring training load to understand fatigue in athletes. Sports Med. 44, 139–147. doi: 10.1007/s40279-014-0253-z 25200666 PMC4213373

[B14] Hopkins W. G. Marshall S. W. Batterham A. M. Hanin J. (2009). Progressive statistics for studies in sports medicine and exercise science. Med. Sci. Sports Exerc. 41, 3–13. doi: 10.1249/MSS.0b013e31818cb278 19092709

[B15] Impellizzeri F. M. Rampinini E. Marcora S. M. (2005). Physiological assessment of aerobic training in soccer. J. Sports Sci. 23, 583–592. doi: 10.1080/02640410400021278 16195007

[B16] Issurin V. B. (2010). New horizons for the methodology and physiology of training periodization. Sports Med. 40, 189–206. doi: 10.2165/11319770-000000000-00000 20199119

[B17] Jauhiainen S. Kauppi J.-P. Leppanen M. Pasanen K. Parkkari J. Vasankari T. . (2021). New machine learning approach for detection of injury risk factors in young team sport athletes. Int. J. Sports Med. 42, 175–182. doi: 10.1055/a-1231-5304 32920800

[B18] Kellmann M. Bertollo M. Bosquet L. Brink M. Coutts A. J. Duffield R. . (2018). Recovery and performance in sport: consensus statement. Int. J. Sports Physiol. Perform. 13, 240–245. doi: 10.1123/ijspp.2017-0759 29345524

[B19] Kiely J. (2012). Periodization paradigms in the 21st century: evidence-led or tradition-driven? Int. J. Sports Physiol. Perform. 7, 242–250. doi: 10.1123/ijspp.7.3.242 22356774

[B20] Laursen P. B. Jenkins D. G. (2002). The scientific basis for high-intensity interval training. Sports Med. 32, 53–73. doi: 10.2165/00007256-200232010-00003 11772161

[B21] Lillicrap T. P. Hunt J. J. Pritzel A. Heess N. Erez T. Tassa Y. . (2015). Continuous control with deep reinforcement learning. arXiv [Preprint]. arXiv:1509.02971.

[B22] McLaren S. J. Macpherson T. W. Coutts A. J. Hurst C. Spears I. R. Weston M. (2018). The relationships between internal and external measures of training load and intensity in team sports: a meta-analysis. Sports Med. 48, 641–658. doi: 10.1007/s40279-017-0830-z 29288436

[B23] Meeusen R. Duclos M. Foster C. Fry A. Gleeson M. Nieman D. . (2013). Prevention, diagnosis, and treatment of the overtraining syndrome: joint consensus statement of the ECSS and ACSM. Med. Sci. Sports Exerc. 45, 186–205. 10.1249/MSS.0b013e318279a10a 23247672

[B24] Plews D. J. Laursen P. B. Stanley J. Kilding A. E. Buchheit M. (2013). Training adaptation and heart rate variability in elite endurance athletes: opening the door to effective monitoring. Sports Med. 43, 773–781. doi: 10.1007/s40279-013-0071-8 23852425

[B25] Puterman M. L. (2014). Markov Decision Processes: Discrete Stochastic Dynamic Programming. Hoboken, NJ: John Wiley & Sons.

[B26] Rein R. Memmert D. (2016). Big data and tactical analysis in elite soccer: future challenges and opportunities for sports science. SpringerPlus 5:1410. doi: 10.1186/s40064-016-3108-2 27610328 PMC4996805

[B27] Rossi A. Pappalardo L. Cintia P. Iaia F. M. Fernandez J. Medina D. (2018). Effective injury forecasting in soccer with GPS training data and machine learning. PLoS ONE 13:e0201264. doi: 10.1371/journal.pone.0201264 30044858 PMC6059460

[B28] Saw A. E. Main L. C. Gastin P. B. (2016). Monitoring the athlete training response: subjective self-reported measures trump commonly used objective measures: a systematic review. Br. J. Sports Med. 50, 281–291. doi: 10.1136/bjsports-2015-094758 26423706 PMC4789708

[B29] Seiler S. (2010). What is best practice for training intensity and duration distribution in endurance athletes? Int. J. Sports Physiol. Perform. 5, 276–291. doi: 10.1123/ijspp.5.3.276 20861519

[B30] Seshadri D. R. Li R. T. Voos J. E. Rowbottom J. R. Alfes C. M. Zorman C. A. . (2019). Wearable sensors for monitoring the internal and external workload of the athlete. npj Digit. Med. 2:71. doi: 10.1038/s41746-019-0149-2 31372506 PMC6662809

[B31] Soligard T. Schwellnus M. Alonso J.-M. Bahr R. Clarsen B. Dijkstra H. P. . (2016). How much is too much? (Part 1) International Olympic Committee consensus statement on load in sport and risk of injury. Br. J. Sports Med. 50, 1030–1041. doi: 10.1136/bjsports-2016-096581 27535989

[B32] Spencer M. Bishop D. Dawson B. Goodman C. (2005). Physiological and metabolic responses of repeated-sprint activities. Sports Med. 35, 1025–1044. doi: 10.2165/00007256-200535120-00003 16336007

[B33] Sutton R. S. Barto A. G. (2018). Reinforcement Learning: An Introduction, 2nd Edn. Cambridge, MA: MIT Press.

[B34] Teixeira J. E. Forte P. Ferraz R. Leal M. Ribeiro J. Silva A. J. . (2021). Monitoring accumulated training and match load in football: a systematic review. Int. J. Environ. Res. Public Health 18:3906. doi: 10.3390/ijerph18083906 33917802 PMC8068156

[B35] Van Hasselt H. Guez A. Silver D. (2016). “Deep reinforcement learning with double Q-learning,” in Proceedings of the AAAI conference on artificial intelligence, Vol. 30 (Phoenix, AZ). doi: 10.1609/aaai.v30i1.10295

[B36] Windt J. Gabbett T. J. (2017). How do training and competition workloads relate to injury? The workload-injury aetiology model. Br. J. Sports Med. 51, 428–435. doi: 10.1136/bjsports-2016-096040 27418321

[B37] Yu C. Liu J. Nemati S. Yin G. (2023). Reinforcement learning in healthcare: a survey. ACM Comput. Surv. 56, 1–45. doi: 10.1145/3477600

